# Changes in the Faculty of the Baltimore College of Dental Surgery

**Published:** 1869-11

**Authors:** 


*Changes in the Faculty of the Baltimore College of Dental Surgery.*—During last month the following changes occurred in the Faculty of this institution : The chair of Chemistry, made vacant by the resignation of Prof. Murdoch, who removed to another city, was filled by the appointment of M. J. De Rosset, M. D., formerly of Wilmington, N. C. but at present Professor of Chemistry in the College of Pharmacy, and also adjunct Professor in the University of Maryland School of Medicine in this city. Professor De Rosset is a practical chemist, having spent years in Europe in the study of his profession, and his appointment will prove very acceptable to the friends of the Baltimore College of Dental Surgery.

Henry Clarke, D.D.S. a graduate of the class of 1859, and formerly associated with Dr. Pleasants of Richmond, Va. in the practice of Dentistry, has been appointed Demonstrator of Operative Dentistry in the place of Dr. H. M. Grant who resigned in September last.

A. G. Bouton, D.D.S. late of Savannah, Geo. and formerly a student of Dr. F. Y. Clarke of Savannah, has been appointed Associate Dental Demonstrator, the College having now three Demonstrators.

The Infirmary of the College having been kept open during the Spring and Summer, there is a considerable increase in the number of patients applying for dental operations.

